# Case Report: Eighteen Month Relapse- Free Survival Following Radical Multidisciplinary Oncological Treatment in a 68-Year-Old Male Patient With Histiocytic Sarcoma

**DOI:** 10.3389/fonc.2021.633215

**Published:** 2021-06-07

**Authors:** Stefan Wehrmann, Hagen Rudolph, Dominikus Ernst, Timo Siepmann, Dorothea Kaltofen, Mathias Hänel, Lutz Mirow

**Affiliations:** ^1^ Department of General and Visceral Surgery, Klinikum Chemnitz gemeinnützige Gesellschaft mit beschränkter Haftung (gGmbH), Chemnitz, Germany; ^2^ Department of Neurology, University Hospital Carl Gustav Carus, Technische Universität Dresden, Dresden, Germany; ^3^ Department of Medical Oncology, Klinikum Chemnitz gemeinnützige Gesellschaft mit beschränkter Haftung (gGmbH), Chemnitz, Germany

**Keywords:** histiocytic sarcoma (HS), surgical oncologic management, multidisciplinary approach, histiocytic and dendritic cell neoplasms, sarcoma – diagnostics, pathology, treatment

## Abstract

**Introduction:**

Histiocytic Sarcoma (HS) is a rare and aggressive malignancy, and patients can present with rapid tumor growth and invasion. The optimal diagnostic and therapeutic management is unknown since only a few cases have been published. Here we report a patient with histiocytic sarcoma of the right groin.

**Case:**

A 68 year-old male patient presented to our hospital with suspicion of a superinfected atheroma of the right groin. Computed tomography showed an abdominal tumor of unknown entity. Detailed assessment including immunohistochemically evaluation of biopsy material confirmed HS. The patient underwent radical tumor resection including compartment-resection of the right thigh. During five additional cycles of chemotherapy over a period of 1.5 years he remained relapse-free.

**Summary:**

Diagnostic work up and treatment of HS is challenging, as there is a paucity of clinical reports and lack of standard guidelines for care. In the present case report, aggressive multidisciplinary treatment resulted in good clinical outcome, however, further studies evaluating this approach in similar patients are needed.

## Introduction

Histiocytic sarcoma (HS) is a rare but aggressive hematopoietic neoplasm with morphologic and phenotypic characteristics of mature tissue histiocytes/macrophages (1). Although more common in male adults the tumor has been reported in all age groups (1–4). HS can occur as an isolated tumor or in association with other hematological neoplasms such as non-Hodgkin lymphoma, acute leukemia or myelodysplasia (5). The histomorphological similarity to other hematological neoplasms like diffuse B-cell lymphomas or peripheral T-cell lymphomas as well as solid tumors like melanoma, other sarcoma and multiple undifferentiated carcinomas impede diagnostics enormously (2, 4, 6). Although gastrointestinal tract, skin, spleen and soft tissue can be affected by the tumor, it is most common in lymph nodes. The cases can be localized or disseminated. Symptoms are frequently unspecific comprising fever, fatigue, night sweats a weight loss. Lymphadenopathy is commonly associated with HS ranging from benign to ulcerating lesions (4).

Immunophenotyping is essential to detect HS. Although there is no specific antibody that shows histiocytic differentiation, diagnosis can be made based on a specific pattern of antibodies viewed in conjunction with the morphology of the tumor hence, HS is a diagnosis of exclusion. In line with this diagnostic challenge, the clinical treatment is equally demanding and has to be multi-disciplinary as HS commonly presents at an advanced clinical stage in which chemotherapy is less effective and mortality is already high. Resection, (neo-) adjuvant chemotherapy or combinations with radiotherapy are most commonly used therapy strategies. Absenting prospective evidence, no standardized treatment guidelines are available to date.

Here we report the course of a 68 year-old male patient with HS of the right groin who has undergone detailed clinical, radiological and immunohistochemically assessment as well as radical multidisciplinary treatment.

## Case

**Figure 1:**
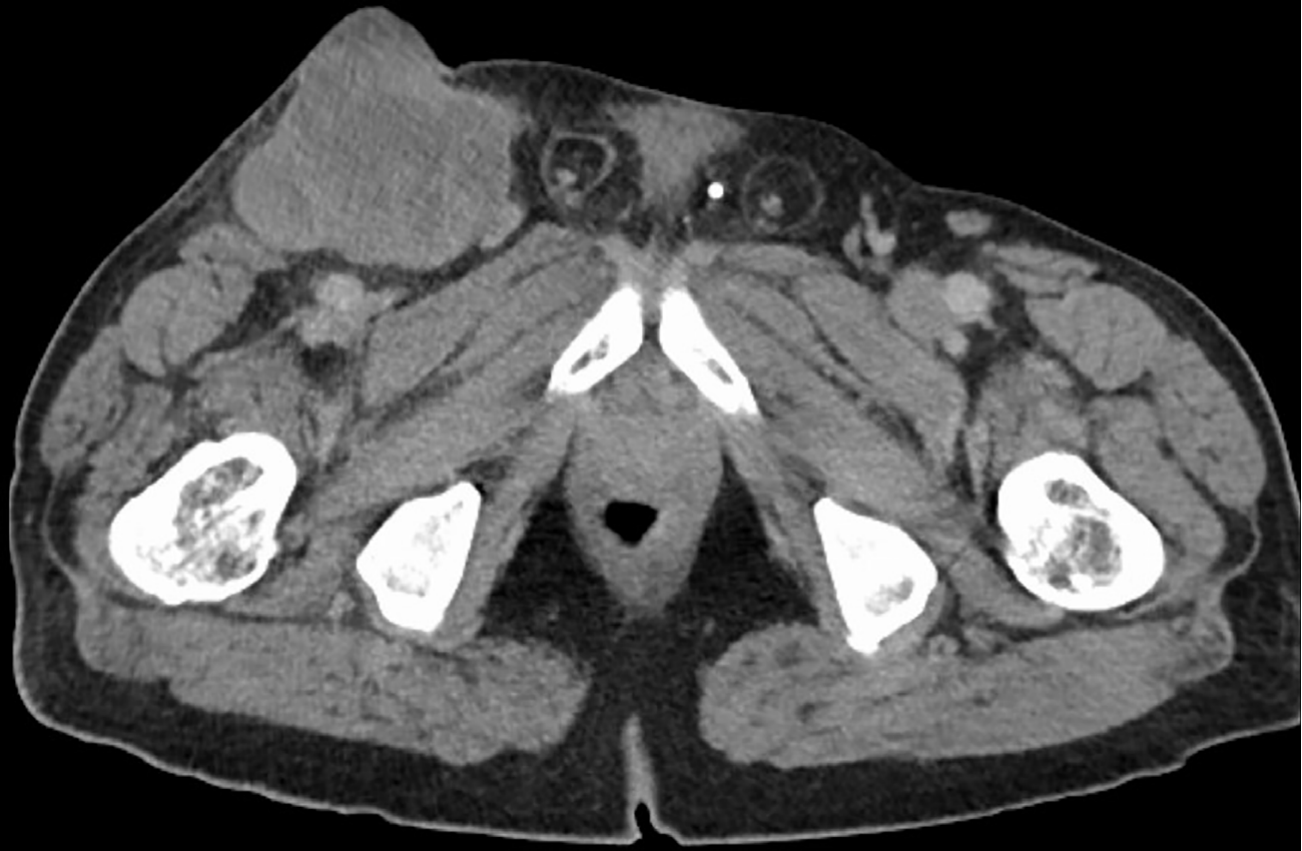
CT of the tumor.

**Figure 2:**
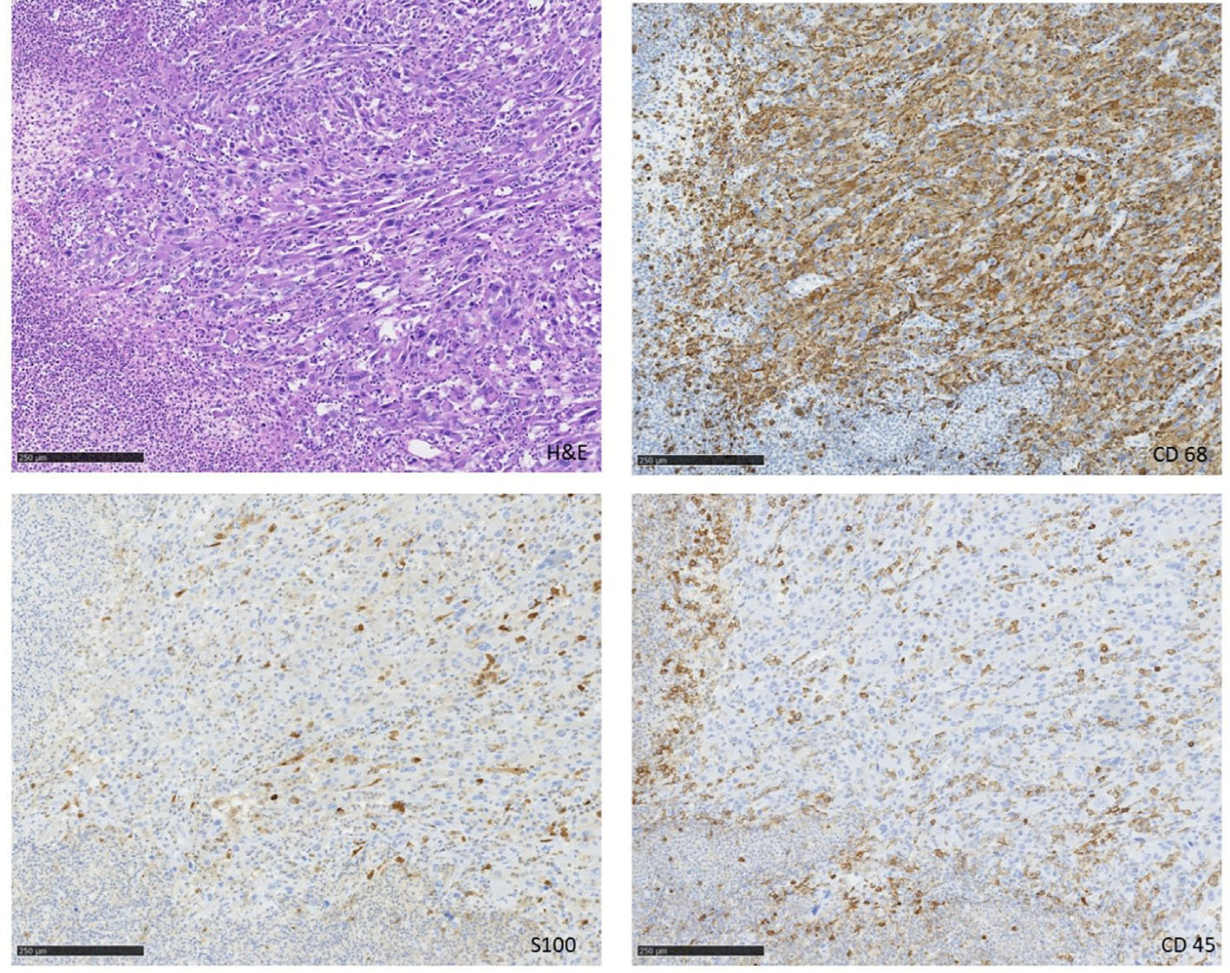
Tumor 15 days after biopsy.

**Figure 3:**
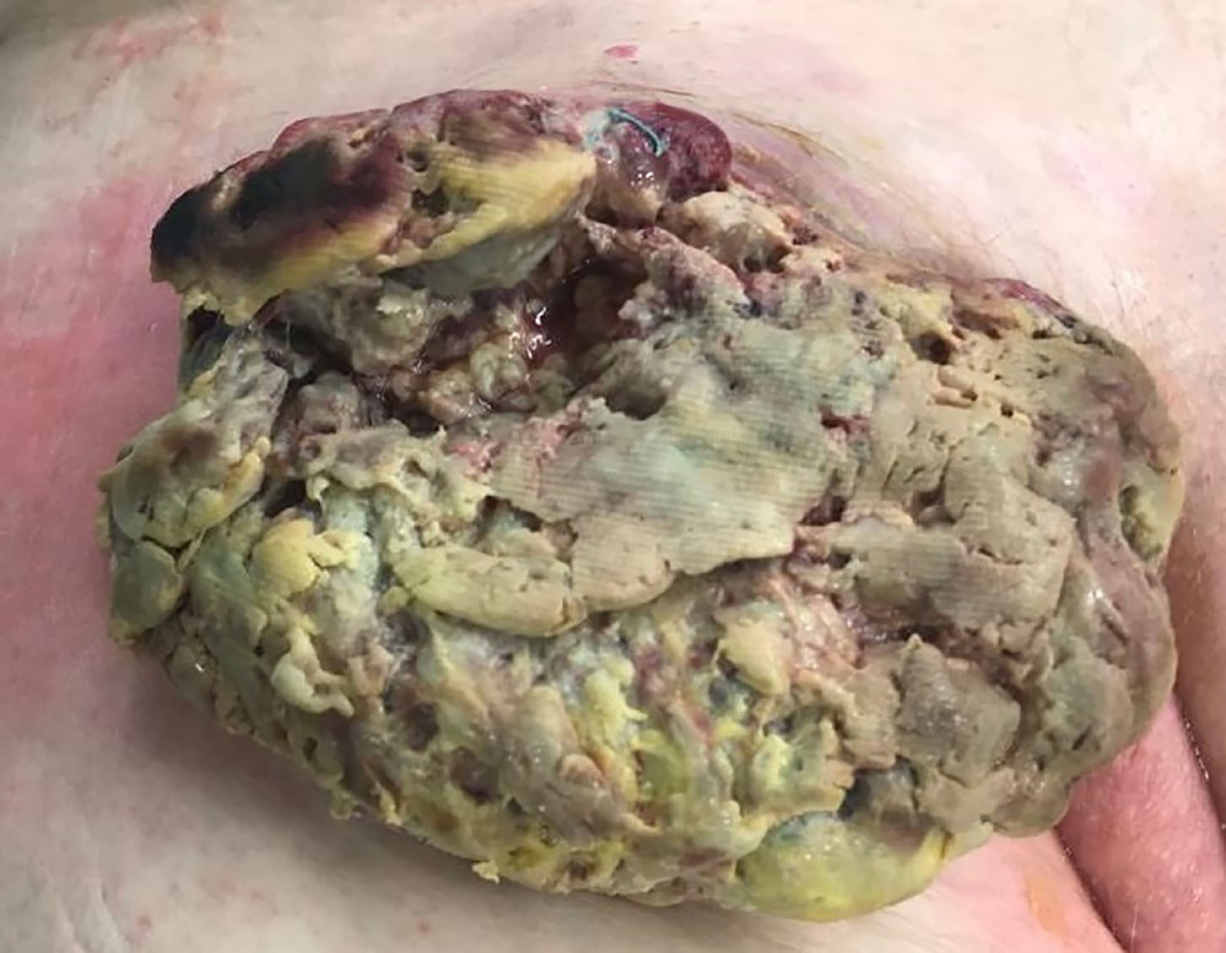
Immunohistochemistry of the tumor.

In July 2019 a 68 year-old patient presented to our emergency department with a painless lesion of the right groin, which has shown growth over the past five months associated with fever, weight loss and night sweats. Initial diagnostic work up displayed elevated infection parameters (C-reactive protein 80 mg/l, leukocyte count 18,8 Gpt/l) and an 8cm-large tumor of the right groin consistent with a lymphatic tumor on computed tomography (**Figure 1**). We then performed an open biopsy of the tumor. Immunohistochemical assessment indicated HS with positive staining for CD 68, S100 and CD 45/protein tyrosine phosphatase (**Figure 2**). Interestingly a subsequent bone-marrow biopsy showed small complexes of HS with an identical immunohistochemical pattern as the primary tumor. After completing the staging diagnostics with bone marrow biopsy, computed tomography of the thorax and abdominal vessels we indicated radically oncological resection of the now ulcerated and grown tumor (**Figure 3**). Fifteen days after hospitalization the tumor doubled its volume.

**Figure 4:**
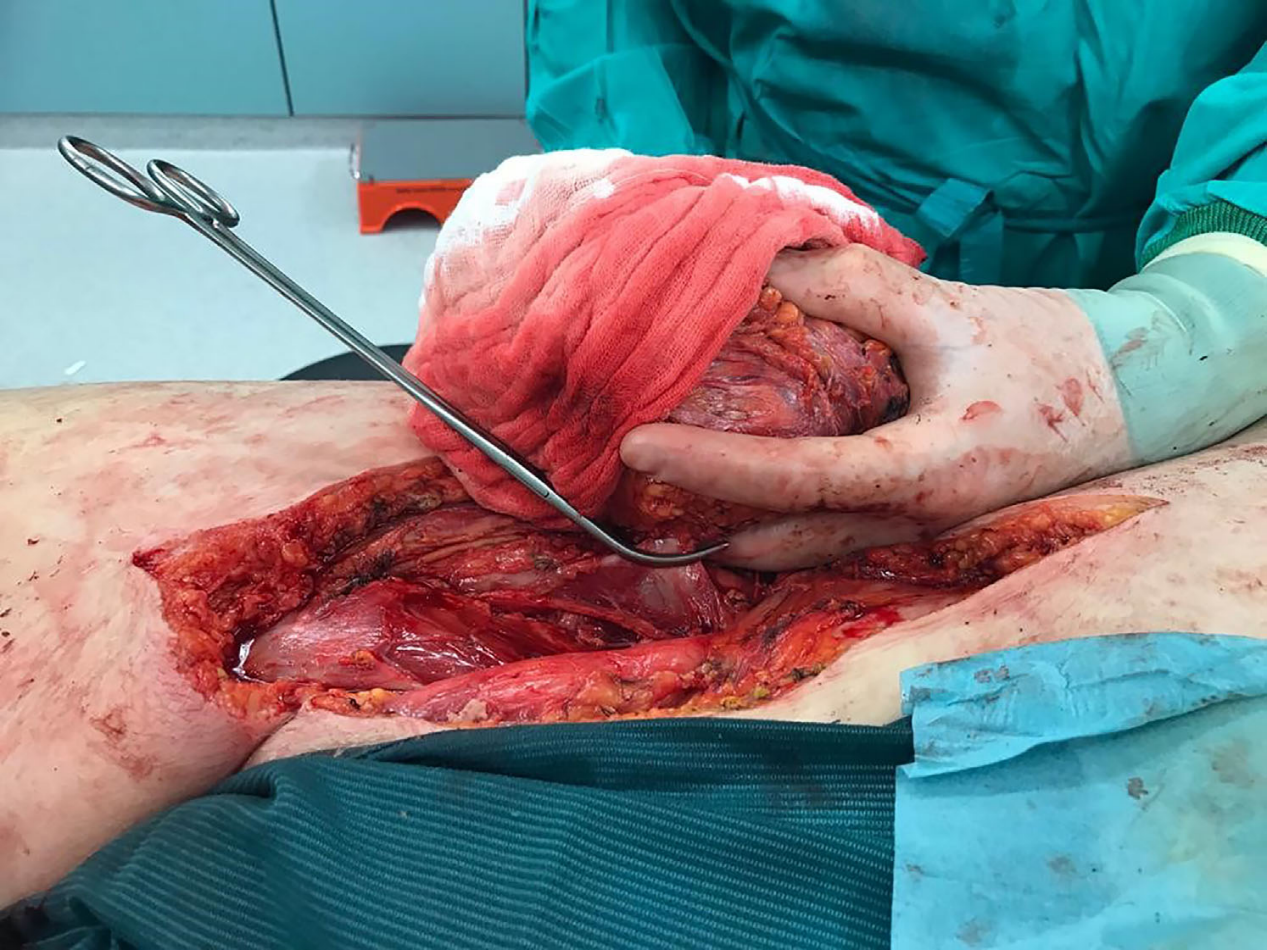
Intraoperative picture.

**Figure 5:**
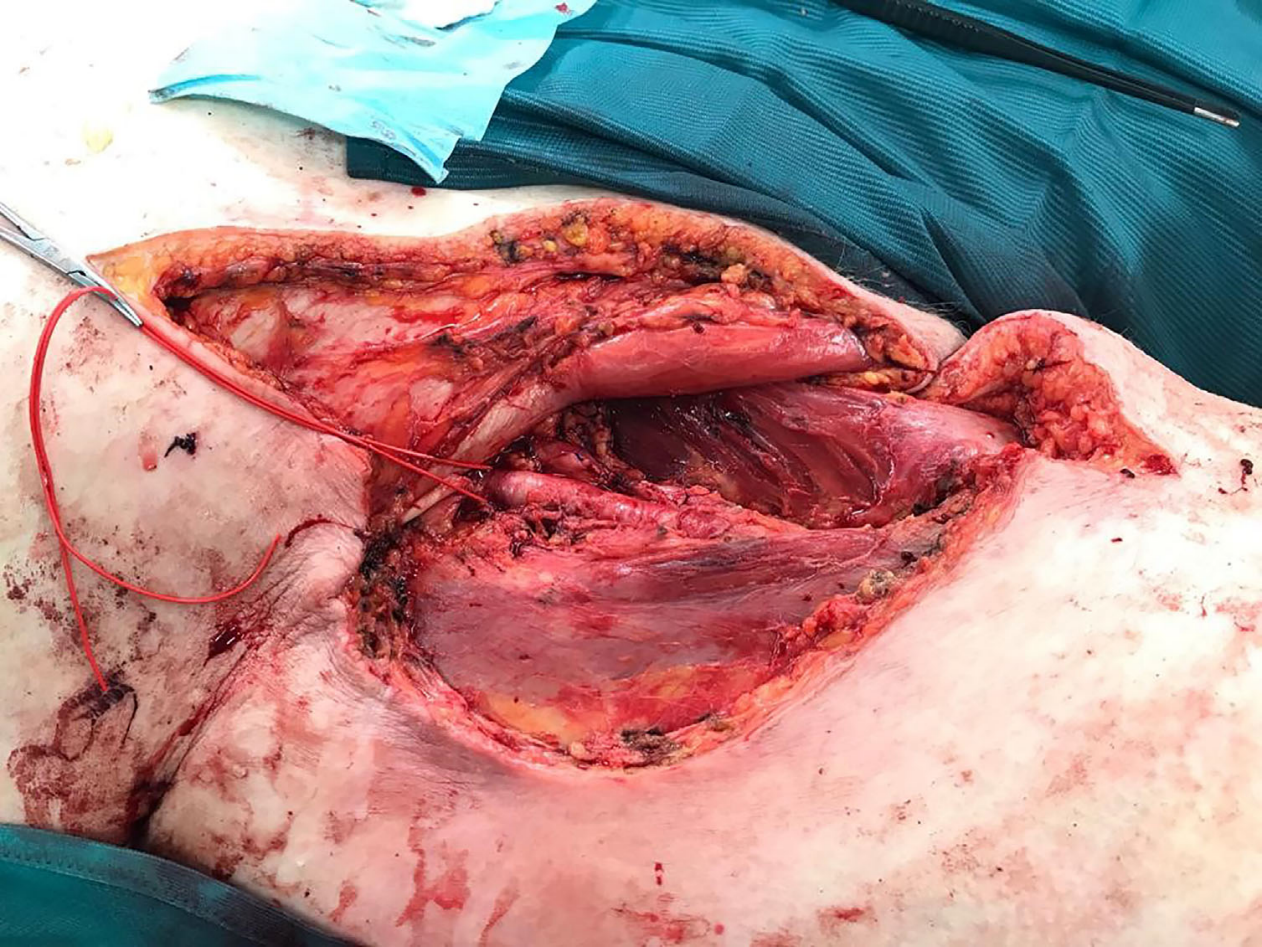
Situs after resection.

**Figure 6:**
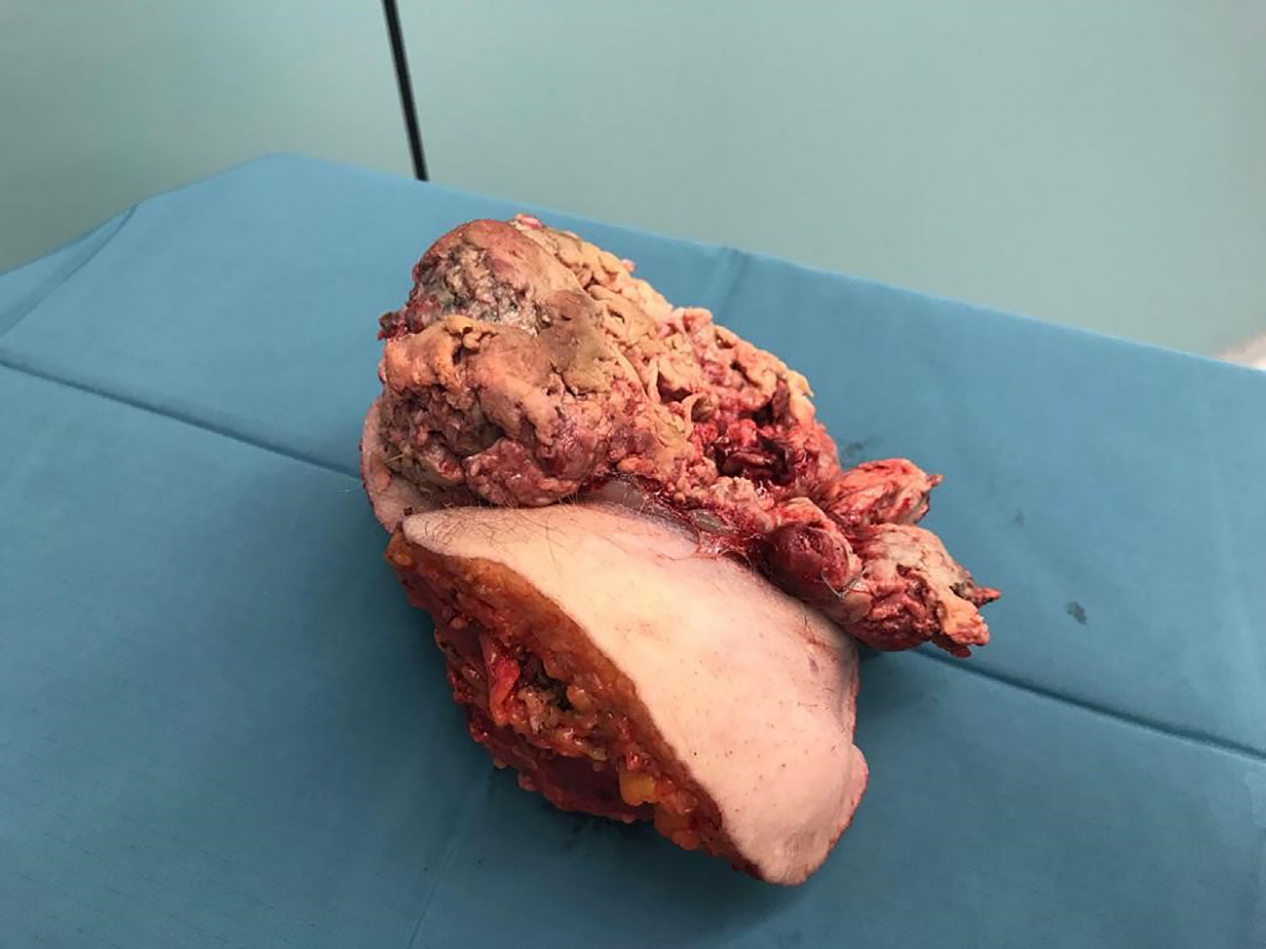
Tumor and compartment.

Because of diameter and infiltrating growth, a compartment-resection of the right thigh with retroperitoneal exposure was needed. Furthermore, we resected tangentially the femoral and external iliac veins. The vessels were covered by a rectus femoris muscle flap (**Figures 4**–**6**). We used vacuseal bandage for primary wound-covering and preferred secondary healing.

After surgery the tumor became examined by our pathologists. Immunohistochemistry and mutational analysis confirmed the diagnosis histiocytic sarcoma with bone marrow-infiltration. Mutation analysis has been realized by using the BIOCARTIS IDYLLA™ automated system. It showed two variants in BRAF, V600E and V600D, in the tumor sample (c.1799T>A/c.1799_1800TG>AA/c.1799_1800TG>AT;c.1799_1800TG>AC.). The final tumor entity was pT4 L0 V0 Pn0 N0 (0/6) M1 (bone marrow) R1, BRAF positive, PD-L1 positive.

Because of this, local and systemic therapy was needed. For adjuvant therapy we preferred intravenous chemotherapy. Hence, we used 5 cycles of CHOEP -scheme (Cyclophosphamide, Hydroxydanuorubicin, Vincristine, Etoposid, Prednisolone). Local radiation would cause disruption in wound healing. Therefore, no other way seemed safe. Nineteen days after surgery we transferred the patient to our department of hematology and oncology.

After 5 cycles of chemotherapy we stopped and did follow up. One and a half year after surgery, the patient was relapse-free. Then a local relapse was diagnosed. He refused another chemotherapy and died one month after.

## Discussion

In our patient with advanced HS of the right groin and satellite tumor lesions in the bone marrow radical surgery and individual chemotherapy led to one and a half years free of relapse.

Tumors derived from histiocytic as well as dendritic cells encompass a large and heterogeneous group of neoplasms. Their diagnosis is challenging both for pathologists and clinicians. Not only the late detection of the tumor, but also the aggressive and fast growth combined with a high rate of local relapse aggravate the decision of therapy. Previous case reports and case series suggested that diagnosis of HS should be supported by imaging including CT and PET-CT followed by bioptic confirmation (5–10). However, the diagnostic value of these assessments for HS detection in poorly understood and requires evaluation in prospective cohorts. In our case, course of the disease suggests that HS has a high risk of fulminant growth. While generalizability of this observation remains unknown, our case substantiates a need for prospective research on HS when viewed in conjunction with previous reports. Hence the alternative of primary resection for large, ulcerating tumors without preoperative histological assessment should be discussed. In order to this, bone marrow biopsies and immunohistochemical staining of the samples, as shown in the case, can help diagnose the tumor without injure the solid tumor.

Tumor-biology is not completely understood. Based on the work of the International Lymphoma Study Group we know that immunohistochemistry seems to be effective to separate HS from the multiplicity of differential diagnosis. Therefore CD68, S100 and Lysozyme seem to be specific (2, 3, 5–7). In doubt a master-program with multiple immunohistochemical stainings could help to find the right diagnosis. In our case all CD68 positive cells were also positive in S100 staining. Furthermore, we know that BRAF-mutation in HS as well as large diameter, elevated lactate dehydrogenase, ECOG performance status and Ann Arbor stage III- IV show a bad prognosis (7).

There are many case reports and reviews on this topic. Unfortunately, because of the rareness of HS structured therapy algorithms do not exist. Even the problem of primary surgery, radiation, chemotherapy or combined therapy is not solved. Many chemotherapy- schemes are reported in former studies. Hence the tendency for using the CHOEP-scheme seems to be effective (6, 8, 9).

In our case, mutational analysis showed two variants of BRAF mutation in Codon 600, V600E and V600D. These mutations likely contributed to the high rate of tumor growth and invasiveness. Previous case reports described key mutations in the MAPK pathway in histiocytic neoplasms (10–12).In a cohort of 28 patients with histiocytic neoplasms, RAS-MAPK pathway mutations were observed in 57% (11). Based on these reports, targeted therapy with the MEK inhibitor cobimetinib might be a promising strategy with relapse free intervals for up to 1.5 years (12). In addition, there are specific BRAF inhibitors, such as vemurafenib (Zelboraf^®^), dabrafenib (Tafinlar^®^), and encorafenib (Braftovi^®^) which could be additional options for such patients in the future if mutation analysis is carried out at the time of surgery, and made part of ongoing treatment after the chemo- and radiation therapy and may extend life (13–15). However, randomized studies to test the possible efficacy of this treatment are lacking to date. Likewise, there is no prospective controlled data to support surgical treatment as the first line therapy of choice. It is noteworthy that in our case analysis of tumor and normal DNA from the patient were not undertaken. This might have allowed further insight in the phenotype of the reported malignancy and should be considered in patients with HS.

Therefore, choosing the first line treatment for histiocytic sarcoma remains a high individual decision that should take into account tumor growth, location, infiltration in vessels or nerves, patients’ co-morbidities, and presence of RAS-MAPK pathway mutation. In many cases, primary surgical therapy followed by a contemporary chemotherapy or MEK inhibitor therapy might be useful strategy. However, prospective well-designed research is urgently needed to test this hypothesis as well as alternative treatment regimens to improve care of patients with histiocytic sarcoma.

Although the overall literature on histiocytic sarcoma is scarce, prognosis seems to be poor with a 6-month mortality of up to 60% (10). In our case, the patient died 1.5 years after surgery. This further substantiates the need for prospective study of targeted treatment strategies that might include mixed approaches comprising both surgery and chemotherapy as well as immunotherapy, e.g. CHOEP and/or BRAF or MEK inhibitors, as well as blocking PD-L1. We recommend that patients with HS should be treated in a specialized sarcoma and cancer center to get an early and sufficient diagnosis and therapy.

## Data Availability Statement

The original contributions presented in the study are included in the article/supplementary material. Further inquiries can be directed to the corresponding author.

## Ethics Statement

Ethical review and approval was not required for the study on human participants in accordance with the local legislation and institutional requirements. The patients/participants provided their written informed consent to participate in this study.

## Author Contributions

SW: Corresponding author. HR, DE, and TS: Preparation of graphical material. DK and MH: Support and arrangement of the publication. LM: initiator. All authors contributed to the article and approved the submitted version.

## Conflict of Interest

The authors declare that the research was conducted in the absence of any commercial or financial relationships that could be construed as a potential conflict of interest.
